# Hahnemann’s Homœopathic Formula

**Published:** 1885-02

**Authors:** Thos. Skinner


### HAHNEMANN'S HOMŒOPATHIC FORMULA.

SIR :—I have been a silent but not an uninterested reader of "the passage at arms" between Drs. Lippe and Dudgeon, and although I have no desire to extend the debate, or to lift the cudgels on either side, I offer the following facts and observations for what they are worth :

Dr. Dudgeon has stated that Hahnemann "always" expressed the formula as *similia similibus curentur*. If there is one translation of the *Organon of Medicine* which I prize more than another, it is Dr. Dudgeon's translation, which was published by Headland, of London, in 1849. This "immortal work"—*vide* translator's preface—is my constant companion in study. I have gone over the entire introduction and the *Organon* itself, purposely to see *how often* Hahnemann used the word *curentur*, or *curantur*. From beginning to end *curantur* never occurs, and on page 55 of "the introduction" only does the term *curentur* appear. It occurs twice in the same page, and is used by Hahnemann, or is wrongly translated by Dudgeon, to express a rule of practice in one instance, where *contraria contrariis curentur* is used, and in another *curentur* is the term used to express "the only therapeutic law conformable to nature—*similia similibus curentur*."

It does appear to me strange that Dr. Dudgeon should use the term "always," and yet in his own translation of Hahnemann's "immortal work," the term *curentur* should only appear once in connection with the law of *similia*, and that once not in the *Organon* itself but in "the introduction" only. In the second place, Dr. Dudgeon has still to prove that Hahnemann actually wrote *similia similibus curentur* as expressing the only law of therapeutics, and not as a mere rule of practice. So far as I am concerned, unless this is found to be the case by an examination of Hahnemann's own manuscripts, I feel in no way bound to believe it. A rule of practice and a law of nature are two very different things, and while *curentur* suits the rule, *curantur* is demanded by an infallible, immutable law.

There may be any number of rules of practice ; there can be only *one law* of cure or of anything else.

Dr. Dudgeon seems to think that it is a matter of no moment which term is used. *Curentur* he used in his translation of "the introduction to the *Organon*," and in his now defunct journal, *The British Journal of Homœopathy*, Dr. Dudgeon has used the universally acknowledged expression of the only therapeutic law, *similia similibus curantur*, and this has for the last forty years been emblazoned on the first page and on the wrapper. Dr. D. may treat this matter with indifference, but where fact and truth are concerned, indifference is criminal—and he must not forget that he is only a translator, though a good one. His translation, nevertheless, is not from the original MS., but only from the fifth German edition of the *Organon*. Let us have it from the original MS. and let it decide.

Nothing was heard of this different reading or rendering of the law until Dr. Carroll Dunham took it up, and he cooked it and offered it as a sop to the "liberty of action" men.

It appeared in the pages of the London *Monthly Homœopathic Review* of February, 1862, and was republished (?) in Dunham's own journal, *The American Homœopathic Review*, of August, 1862. It may be difficult to reconcile these dates, but it is fact nevertheless. As I do not possess the *Monthly Homœopathic Review* for 1862, I think your readers will be interested to know what Dr. Dunham's views in this matter were from the pages of his own journal. They were as follows:

"To think is to *thing;* a thought is a thing (I always believed and do still believe that a thought was a thing thought, and not a thing); words are the exponent of the thoughts of speech, articulate man. Those who do not know the value of words can have but a very imperfect notion of things. Men, not thinkers, are governed by the words of those who do think [Good! hence the numbers who are led astray], and in most of the quarrels about matters dogmatically treated the differences result from the misapprehensions of the meaning of words.

"A dispute about a diphthong once caused disastrous wars and still influences theology. * * * * *

"Some grand truths are instinctively held, or perceived instinctively, as it were; others loom on the mind gradually and are brought nearer and nearer, as the peak of Teneriffe first looks like a cloud as big as a hand, then gradually grows as the voyager approaches.

"'A thing of beauty is a joy forever,' and there is no beauty like that of naked truth, stripped of all fig-leaves and pigment, simple, sincere, severe in God-like purity and majesty.

"We homœopathists hold that our law, *similia similibus curentur*, is such a truth. The nearer you approach it, the more beautiful it is; touch it, grasp it, cherish it. The mistakes about things from mistakes about the words used to signify those things has been adverted to. The very law of Homœ-

opathy has been subjected to false and vicious interpretations *by substituting one letter, one vowel, for another, a for e.* [The italics are mine.—Tho. S.] Hahnemann was a good though, in a critical sense, not a profound scholar. The old hero knew very well the value of the words he employed. He was incapable of the ridiculous solecism, of the *ignorance* which is perpetuated on the title-page of *The British Journal of Homœopathy.* His expression for this law of drug-healing was and is *similia similibus curentur*, not *curantur.*

"His best beloved friend and his reverend pupil, the late Rev. T. R. Everest, told us how much Hahnemann was annoyed at the employment of the word *curantur.*

"In the medical sense the Latin verb *curo* means to take care of, to treat, to *doctor.*" (According to Ainsworth, it also signifies "*to cure or heal,*" hence our word *cure*—and Ainsworth quotes Cicero in proof of this—"*Adolescentes gravius ægrotant, tristius* CURANTUR."—Tho. S.)

Dr. Dunham continues to state: "Hahnemann was too much of a philosopher to arrogate the cure; he proposed the *treatment.* 'Let likes be *treated* by likes' [the italics in treated are mine. —Tho. S.], that is the formula or expression for the law of drug-healing. In that formula he expresses *one* of Nature's laws of healing—that is, a law of God; the expression foisted on him is an impertinence. Let this formula be adopted: *Similia similibus curentur.*" (The word "one" is italicized by myself.—Tho. S.)

Dr. Dunham further adds: "In the second edition of the *Organon*, in Dresden, 1819, the formula is given *similia similibus curentur*, Introduction, p. 29. In the British translation of the fourth German edition of the *Organon*, and which was reprinted in New York, 1843, with a preface by Dr. Hering, the same reading is given.

"In the *British Journal of Homœopathy*, Vol. I, Introduction, and subsequently it is written *similia similibus curantur.*

"In *The Homœopathic Examiner*, Vol. I, p. 25, it is given by Dr. Hull *similia similibus curantur.*" "DUNHAM."

(From *The American Homœopathic Review*, August, 1862, pp. 36, 87, edited by Drs. P. P. Wells, Carroll Dunham, and Henry M. Smith.)

I may be wrong, and no one knows better than myself that when I first joined the ranks of Homœopathy I was fairly taken in by the above plausible special pleading of Dr. Dunham. I was one of those who, from insufficiency of thought, "are governed by the words of those who do think," and in my journal, the *Organon*, I acknowledge to have been misled by this very article of the shrewd and calculating Dr. Dunham. Only fancy! the difference between the use of the letter *e* for *a* reduces the only law of therapeutics into a rule of practice in drug-treatment. *Curentur* and *curantur* have no relation to the cure of disease by one fixed law of nature, or the healing of the sick. The Latin verb *curo*, Dr. Dunham tells us, signifies "to take care of, to treat, to doctor"—not to cure or heal.

The Latin word *curo*, besides signifying "to take care of, to treat, to doctor," undoubtedly signifies to "*cure or heal*," *vide* Ainsworth's large quarto Latin Dictionary, the best authority in existence. I do not know, nor do I care to know, where Dr. Dunham got the signification, "to treat, to *doctor*," certain it is that among the fourteen or fifteen different significations of the verb *curo* he will not find these in Ainsworth; and to "doctor" he has given in italics. Our word *cure* comes direct from *curo*, and from no other source, and so does our term *curate* and *cure*, in French, signifying the carer, or curer of souls. No, the true signification of the verb *curo*, to cure or heal, of Ainsworth and of all who know anything of Latin, and, like Hahnemann, not "profound scholars," did not suit Dr. D.'s purpose, so he purposely left them out, at least, so it appears.

Let us not forget that while he, Dr. D., admits *the law* of drug-healing to be expressed by the formula, "Let likes be *treated* by likes," he contradicts himself in the next breath by stating that the formula "expresses *one* of Nature's laws of healing, that is, a law of God." Then God has more *laws* than one of therapeutics, that is, of healing the sick! I wonder how many He has of gravitation? "Let likes be *treated* by likes" is the expression or formula of no law; it can amount to no more than "a rule of the road," but, that diseases are cured and can only be cured by drugs capable of inducing in the healthy the most similar diseased conditions that are found in the sick, which is clearly and succinctly expressed by the formula, *similia similibus curantur*, no honest man who has faithfully and honestly practiced medicine as Hahnemann directs will ever doubt, Drs. Dunham, Dudgeon, Drysdale, and Hughes to the contrary notwithstanding.

We want no more quotations from print; let us have the manuscript of Hahnemann, and no more of his second-hand translators. Even supposing Hahnemann to have written *curentur* instead of *curantur*, nothing short of *curantur*—likes *are* cured by likes—would make it *a law of cure*, *curentur* going no further than *may be a mere rule of practice.*

In conclusion, let me make the following quotation from Dr. Mayne's Medical Lexicon, two volumes, Churchill, 1860. Dr. Mayne is our highest authority as a medical lexicographer, and he gives the formula of Hahnemann (Vol. II) as *similia similibus curantur*, and at page 281 of Vol. I, under the rubric *Doctrine of Signatures*, he has the following interesting historical record: "Certain plants and medicinal agents were believed to be so marked or stamped that they presented outwardly or visibly the indications of the diseases or diseased organs for which they were specifics; these were their *signaturæ*. Hence, anciently, the proper specific for a disease was learned and determined by ascertaining what plants in their usual properties were *similar or analogous to the predominating symptoms of disease* or to the organs diseased; *similia similibus curantur*. To this doctrine we owe some popular names of plants, as eye-bright, liver-wort, spleen-wort, etc."

In the new edition of *Mayne's Medical Lexicon*, now in course of publication by the "New Sydenham Society," the *Doctrine of Signatures* is considerably altered, if not mangled, all reference to "similar or analogous symptoms," etc., and the law of *similia similibus curantur* being *non est inventus*. Of course, this is no fault of Dr. Mayne's, but of the Society and the present editors, who all belong to the old school. Instead of *altering a vowel* they prefer to *expunge the whole law*.

I repeat, I send you these cursory remarks for what they are worth, and if they only lead some one to examine Hahnemann's manuscript, I shall not have written them in vain.

THOS. SKINNER, M. D.

